# Comment on Li et al. Association of Triglyceride–Glucose Index and Coronary Chronic Total Occlusion in Patients Undergoing Coronary Angiography: A Retrospective Study. *J. Cardiovasc. Dev. Dis.* 2026, *13*, 275

**DOI:** 10.3390/jcdd13080351

**Published:** 2026-07-28

**Authors:** Güney Sarıoğlu, Yakup Yiğit, Abdulmecit Afşin

**Affiliations:** Department of Cardiology, Ministry of Health, Battalgazi State Hospital, 44320 Malatya, Turkey; ygtykp@gmail.com (Y.Y.); abdulmecit.afsin@ozal.edu.tr (A.A.)

We read with interest the study by Li et al. [1] describing the independent association of the triglyceride–glucose (TyG) index with chronic total occlusion (CTO) in 1157 patients undergoing coronary angiography, with a non-linear dose–response relationship and an independent association after further adjustment for diabetes. This study is relevant as an extension of prior CTO-based research, which posits that higher TyG is associated with a more severe coronary phenotype and an adverse prognosis [1]. Although TyG reflects insulin resistance and combined glycaemic–lipid burden, CTO development is biologically more complex. Chronic inflammation, endothelial dysfunction, oxidative stress, progressive plaque accumulation, plaque disruption, thrombosis, and thrombus organisation may all contribute to the transition from atherosclerotic stenosis to chronic occlusion. Insulin resistance may amplify these processes by reducing nitric oxide bioavailability, increasing endothelial activation and inflammatory signalling, and promoting platelet hyperreactivity and a prothrombotic state. Therefore, TyG should be interpreted as an indirect marker of a metabolically adverse vascular environment rather than as a direct mechanistic marker of CTO. Integrating TyG with inflammatory biomarkers and plaque-imaging characteristics may provide a more biologically complete risk assessment. This concept is also supported by evidence from other high-risk coronary populations. In patients with ST-elevation myocardial infarction undergoing primary percutaneous coronary intervention, the Advanced Lung Cancer Inflammation Index, a composite marker incorporating inflammatory and nutritional status, was associated with all-cause mortality. Although these findings cannot be directly extrapolated to CTO, they reinforce the broader principle that integrating metabolic, inflammatory, and nutritional indices may provide more comprehensive prognostic information than relying on a single metabolic marker such as TyG. More broadly, persistent systemic inflammation is increasingly recognised as an important contributor to the progression and adverse outcomes of chronic cardiovascular disease, including chronic heart failure. Although evidence from heart failure populations cannot be directly extrapolated to CTO, it supports the concept that metabolic dysfunction and chronic inflammation may interact throughout cardiovascular disease progression. Within this framework, TyG may reflect one component of an adverse metabolic–inflammatory milieu but should not be regarded as a direct measure of vascular or systemic inflammation [2,3,4].

However, there is another, more significant aspect beyond recognised design limitations when interpreting this association in the context of shape-of-risk and treatment confounding. The authors describe the non-linear TyG-CTO relationship; however, the previous literature on CAD risk based on broad meta-analysis has shown the opposite—i.e., linear association, while in different coronary populations, U-shaped non-linear association of TyG with the incidence of major adverse cardiovascular events above the inflexion point near 8.62–8.77 has been found [5,6].

Thus, TyG does not seem to be a universal, monotone marker, and the threshold effect should be discussed before considering it as an independent CTO-related biomarker. In patients already referred for coronary angiography, the clinical interpretation of such non-linearity differs from that in population-based screening settings. These patients constitute a selected, high-risk population with a high pre-test probability of coronary artery disease; therefore, TyG should not be considered a stand-alone screening or diagnostic tool. Instead, a non-linear association indicates that the incremental probability of CTO associated with TyG may differ across its distribution, potentially becoming more apparent only beyond a population-specific inflexion point. Consequently, an identical increase in TyG should not be assumed to convey an equivalent increase in CTO probability at all baseline values. Any proposed threshold should therefore be externally validated and interpreted alongside clinical presentation, angiographic characteristics, diabetes status, and lipid- and glucose-lowering therapies.

Li et al. [1] appropriately acknowledge both the absence of detailed lipid-lowering medication data and the limited discriminatory performance of the TyG index. We further emphasise that these limitations have important clinical implications. Lipid-lowering therapies, particularly statins, may adversely affect glycaemic control and insulin resistance, thereby influencing TyG independently of the underlying metabolic burden, leaving the possibility of residual treatment-related confounding [7,8]. Moreover, the reported AUC of 0.556 indicates only marginal discrimination beyond chance. Thus, although TyG may be statistically associated with CTO at the population level, it cannot reliably classify individual patients or be considered a stand-alone diagnostic criterion for CTO. Any proposed clinical application should require evidence that TyG adds meaningful discrimination, calibration, or decision benefit beyond established clinical and angiographic variables. This distinction between statistical association and discriminatory performance is particularly important because the prognostic contribution of TyG appears to be context-dependent and may vary according to baseline cardiovascular risk and the presence of established cardiovascular disease [9]. Importantly, statistical association should not be conflated with diagnostic performance. Although TyG may be independently associated with CTO, the reported AUC of 0.556 indicates only weak discrimination and does not support its use as a stand-alone diagnostic criterion or screening tool. Therefore, the principal clinical message is that TyG may reflect metabolic risk at the population level, but its value for classifying individual patients with respect to CTO is limited. Any proposed clinical application should require evidence that TyG adds meaningful discrimination, calibration, or decision benefit beyond established clinical and angiographic variables. This distinction between causal association and discriminatory power is very important, especially given the prior literature on the prognostic value of TyG, which is context-dependent and sometimes attenuated in the presence of established cardiovascular disease [9].

In conclusion, the available evidence suggests that TyG may reflect an adverse metabolic milieu associated with CTO, but its clinical interpretation is limited by potential non-linearity, treatment-related confounding, modest discriminatory performance, and the multifactorial pathophysiology of chronic coronary occlusion. TyG should therefore not be regarded as a stand-alone diagnostic criterion or as a direct mechanistic biomarker of CTO. Future studies should employ adequately powered, longitudinal, multicenter designs with standardised CTO definitions, comprehensive adjustment for lipid-lowering, glucose-lowering, antithrombotic, and other relevant cardiovascular therapies, and repeated TyG measurements to capture temporal variability and cumulative metabolic burden. Such studies should also assess whether TyG provides incremental value beyond established clinical, angiographic, inflammatory, and metabolic markers and should prioritise hard cardiovascular outcomes, including cardiovascular death, myocardial infarction, stroke, heart-failure hospitalisation, and unplanned coronary revascularisation.

## Data Availability

No new data were created or analyzed in this study. Data sharing is not applicable to this article.
